# Detection of tactile-based error-related potentials (ErrPs) in human-robot interaction

**DOI:** 10.3389/fnbot.2023.1297990

**Published:** 2023-12-12

**Authors:** Su Kyoung Kim, Elsa Andrea Kirchner

**Affiliations:** ^1^Robotics Innovation Center, German Research Center for Artificial Intelligence GmbH, Bremen, Germany; ^2^Institute of Medical Technology Systems, University of Duisburg-Essen, Duisburg, Germany

**Keywords:** human-robot interaction, implicit error detection, error-related potentials, orthosis, EEG, tactile-based ErrP detection

## Abstract

Robot learning based on implicitly extracted error detections (e.g., EEG-based error detections) has been well-investigated in human-robot interaction (HRI). In particular, the use of error-related potential (ErrP) evoked when recognizing errors is advantageous for robot learning when evaluation criteria cannot be explicitly defined, e.g., due to the complex behavior of robots. In most studies, erroneous behavior of robots were recognized visually. In some studies, visuo-tactile stimuli were used to evoke ErrPs or a tactile cue was used to indicate upcoming errors. To our knowledge, there are no studies in which ErrPs are evoked when recognizing errors only via the tactile channel. Hence, we investigated ErrPs evoked by tactile recognition of errors during HRI. In our scenario, subjects recognized errors caused by incorrect behavior of an orthosis during the execution of arm movements tactilely. EEG data from eight subjects was recorded. Subjects were asked to give a motor response to ensure error detection. Latency between the occurrence of errors and the response to errors was expected to be short. We assumed that the motor related brain activity is timely correlated with the ErrP and might be used from the classifier. To better interpret and test our results, we therefore tested ErrP detections in two additional scenarios, i.e., without motor response and with delayed motor response. In addition, we transferred three scenarios (motor response, no motor response, delayed motor response). Response times to error was short. However, high ErrP-classification performance was found for all subjects in case of motor response and no motor response condition. Further, ErrP classification performance was reduced for the transfer between motor response and delayed motor response, but not for the transfer between motor response and no motor response. We have shown that tactilely induced errors can be detected with high accuracy from brain activity. Our preliminary results suggest that also in tactile ErrPs the brain response is clear enough such that motor response is not relevant for classification. However, in future work, we will more systematically investigate tactile-based ErrP classification.

## 1 Introduction

In recent years, the human-in-the-loop approach has shown great impact on human-robot interaction, and the effects of both explicit and implicit evaluation in robot learning in human-robot interaction have also been well-studied (Daniel et al., 2014; Iturrate et al., 2015; Kim et al., 2017, 2020a,b). In particular, implicitly extracted error detection, e.g., by using electroencephalogram (EEG) is advantageous for robot learning, when evaluation criteria cannot be explicitly defined, e.g., due to the complex behavior of robots or subjective preferences of the interacting person (e.g., Kim et al., 2017).

For detection of implicit evaluation of errors, error-related potentials (ErrPs) have been widely used for various applications (see review, Chavarriaga et al., 2014). In the literature, ErrPs have been classified into four types: response ErrPs (ErrPs elicited by self-induced errors; Falkenstein et al., 2000), feedback ErrPs (ErrPs elicited when recognizing errors after receiving the feedback indicating erroneous events; Holroyd and Coles, 2002), observation ErrPs (ErrPs elicited when observing erroneous actions; van Schie et al., 2004), interaction ErrPs [ErrPs elicited during interaction (Ferrez and Millán, 2008), and i.e., originally interface errors caused by interaction with BCI interfaces].

In robotics, ErrPs have been evoked when observing erroneous actions of robots or systems (Iturrate et al., 2010; Kim and Kirchner, 2013, 2015) or interaction with robots (Kim et al., 2017, 2020a). In human-robot interaction, ErrPs have been used for robot learning (Iturrate et al., 2015; Kim et al., 2017), co-adaptation (Ehrlich and Cheng, 2018), or corrections of erroneous robot behavior (Salazar-Gomez et al., 2017). Furthermore, ErrP-based error detections have been widely used for adaptive classifier of P300 or motor imagery, e.g., by correcting misclassification of P300 (Dal Seno et al., 2010; Combaz et al., 2012; Margaux et al., 2012) or motor-related cortical potentials (MRCPs; Bhattacharyya et al., 2017; Tao et al., 2023) in brain-computer interface (BCIs) area.

In most ErrP-based studies, ErrPs were elicited when recognizing errors visually. For example, ErrPs were elicited when subjects recognized erroneous behavior of robots while observing (e.g., Iturrate et al., 2010; Kim and Kirchner, 2013) or interacting with robots (e.g., Kim et al., 2017). However, tactile-based ErrP classifications have not been widely investigated. Although tactile-based BCIs are not studied as intensively as visual-based BCIs, tactile-based BCIs have a potential for motor rehabilitation and exoskeleton control in rehabilitation applications (e.g., Yao et al., 2022; Lakshminarayanan et al., 2023). In ErrP-based studies, some studies investigated error detections using visuo-tactile stimuli. For example, visual and tactile stimuli were used together to detect errors and these visuo-tactile stimuli evoked ErrPs (Tessadori et al., 2017; Schiatti et al., 2019). In other studies, tactile stimuli were used to indicate upcoming errors, but the errors were recognized visually. Thus, ErrPs were evoked by visual recognition of errors indicated by the tactile cue (Chavarriaga et al., 2012; Ahkami and Ghassemi, 2021). In Perrin et al. (2008), various modalities of stimuli (visual, auditory, tactile) were compared, in which the effect of different modalities of stimuli on reaction time was systematically investigated, but only preliminary results of ErrP classification were shown due to the very small sample size and very large variability between subjects and between stimulus types. In this study, the modalities of stimuli (visual, auditory, tactile) were used to indicated upcoming errors, and the visual recognition of errors evoked ErrPs. In summary, to our knowledge, there were no studies in which ErrPs were evoked when recognizing errors only through tactile channel. In Section 1.1, ErrP-based BCI studies using visuo-tactile stimuli are described in detail.

### 1.1 Related works

In Tessadori et al. (2017), both visual and tactile stimuli were used together, in which the subjects received a vibration from the wristband (Myo armband) in two of three experiments. Here, the subjects wore a Myo armband on each of their left and right wrists. ErrPs were not evoked when a cursor (green square) moved to the target position (orange square), whereas ErrPs were evoked when the cursor moved in the opposite direction of the target position. The authors hypothesized that the additional use of tactile stimuli would increase ErrP classification performance. In the first experiment, errors were recognized visually without the use of tactile stimuli. In the second experiment, the Myo wristband vibrated according to the direction of the cursor movements, e.g., the Myo armband of the right wrist vibrated when the cursor move to the right or vice versa. In this case, the visual recognition of the cursor movement was congruent with the tactile stimulation. In the third experiment, the Myo armband vibrated not according to the direction of the cursor movements, e.g., the cursor moved to the target position (move to the right), but the Myo armband of the opposite site of the target vibrated (vibration of the Myo armband on the left wrist). That means, the visual recognition of the cursor movement was not congruent with the tactile stimulation. The authors found that classification performance was higher when both visual and tactile stimuli were used together than when only visual stimuli were used (first vs. second experiment or first vs. third experiment). However, there was no difference in classification performance depending on congruency of visual and tactile stimuli caused by a mismatch between the two types of feedback (second vs. third experiment).

In Schiatti et al. (2019), visuo-tactile stimuli were used to evoke ErrPs. ErrPs were not evoked when the cursor moved in the direction depicted by the arrow which indicated the correct direction (e.g., move to the left). When the cursor did not move in the direction pointed by the arrow (e.g., move to right, up, or down), ErrP were evoked. ErrP classification performance was slightly higher when visual and tactile stimuli were used together than when only visual stimuli were used.

In Chavarriaga et al. (2012), a visual (arrow) or tactile stimulus (vibration) was used to indicate the upcoming movement direction of the simulated robot, and ErrPs were evoked during visual recognition of the incongruence between the (visually or tactilely) cued movement direction of the simulated robot and the direction of the actually executed movement of the simulated robot. The authors performed two experiments (visual cue and tactile cue). In the first experiment, a visual cue was used to indicate the upcoming movement direction of the simulated robot (i.e., visually presented arrow). ErrPs were elicited during visual recognition of the incongruence between the visual cue and the direction of the actually executed movement of the simulated robot. In the second experiment, a tactile cue was used to indicate the upcoming movement direction of the simulated robot (vibration instead of visual arrow). ErrPs were elicited during visual recognition of the incongruence between the tactile cue and the direction of the actually executed movement of the simulated robot. In both experiments, the incorrectly chosen movement direction of the simulated robot (errors) was recognized visually and ErrPs were evoked when recognizing these errors.

In Ahkami and Ghassemi (2021), a visual or tactile stimulus was used as a cue to indicate the upcoming movements or the direction of upcoming movements. ErrPs were evoked during the recognition of (1) the incongruence between the cued movements and the absence of movements (i.e., errors) or (2) the incongruence between the cued movement direction and the direction of the actually executed movement (i.e., error). The correct and incorrect condition were predefined depending on movement direction or absence of movement: it was correct if the red square moved to the left (first, third, and fourth experiment), whereas it was incorrect when the red square moved to the right (first, third, and fourth experiment) or when the red square did not move at all (second experiment), indicating that there was no correct condition in the second experiment. In the first experiment, ErrPs were evoked when the red square moved to the right (incorrect condition) and ErrPs were not evoked when the red square moved to the left (correct condition), in which the green and red squares were visually presented. That is, the visual recognition of errors can evoke ErrPs. In the second experiment, according to the authors only tactile stimulation was used to evoke ErrPs. Here, the subjects were told that the red square would move after tactile stimulation and they observed the red square visually. That is, the tactile stimulation was used as a cue to anticipate *the movement* of the red square, but the errors (i.e., the absence of the movement of the red square) were visually recognized. Thus, visual recognition of the absence of movement of the square (error) did elicit ErrPs, not the recognition of tactile stimulation. In the third and fourth experiments, tactile stimulation was used as a cue to anticipate the *movement direction* of the red square (i.e., the red square moves to the right or left). If the direction of movement indicated by tactile stimulation (e.g., move to the right) does not match the visual recognition of the direction of movement of the red cursor (e.g., move to the left), ErrPs can be elicited.

In Perrin et al. (2008), six different types of stimulus used to indicate errors were compared in a simulated robot control: visual arrows, visual cursors, auditory tones, auditory words, and vibro-tactile actuator. Subjects were asked to press a button when recognizing error (i.e., incongruence between the visually, auditorily, tactilely cue and the executed action of the simulated robot). Here, the action of the simulated robot was visually recognized. The reaction time was shorter for visual stimuli (visual arrows, visual cursors) than auditory stimuli (auditory tones, auditory words) or tactile stimuli. There was no significant difference in reaction time between auditory and tactile stimuli, but the reaction time was longer for auditory stimuli than tactile stimuli. Especially, the voice cue had the longest reaction time and the largest variation between subjects. ErrP classification performance was very heterogeneous between subjects and between stimulus types so that the comparison between six different types of stimulus was not possible, e.g., the ranking of ErrP classification performance depending on stimulus types was completely different between subjects. Behavioral data (e.g., reaction time) was collected from twenty two subjects, but EEG data was recorded only from four subject of them. For this reason, the authors noted that the results of ErrP classification were preliminary.

In summary, visual and tactile stimuli were used together to evoke ErrPs (Tessadori et al., 2017; Schiatti et al., 2019) or tactile stimuli were used to indicate upcoming errors and the visual recognition of errors evoked ErrPs (Perrin et al., 2008; Chavarriaga et al., 2012; Ahkami and Ghassemi, 2021).

### 1.2 Approaches and goals

In our study, we used tactile stimuli directly to evoke ErrPs without the combination with the visual channel. We also did not use any other cues (visual, auditory, or tactile cue) to indicate upcoming errors. We aimed to evoke ErrPs when recognizing errors only through tactile channel.

In our scenario, the subjects wore an orthosis on their right arm and performed arm movements (details, see Section 2.2). Sometimes, the orthosis did not work correctly, i.e., the orthosis briefly moved in the opposite direction of the intended movements of subjects. This malfunction of the orthosis was preprogrammed to induce errors. Here, we expected ErrPs when subjects recognized such malfunction of the orthosis (i.e., interaction errors) only through the tactile channel.

Since we did not find studies in which ErrPs were evoked when tactile errors were recognized, we asked the subjects to press a button when they felt the malfunction of the orthosis to ensure that the subject can detect tactile based errors. However, in our previous studies (Kim et al., 2017, 2020a,b), we have successfully classified ErrPs without motor response (e.g., button press).

On the other hand, we assumed that the motor potential can also be used for feature extraction of ErrP classification because the latency between the occurrence of errors and the response to errors (e.g., button press) was short. Therefore, we performed additional tests, i.e., test experiments, in a scenario, in which a motor response was not requested after error detection to better interpret our results and to ensure that ErrP-classification performance might be increased or decreased, e.g., without motor response (see **Figure 3** and **Table 5**). To this end, we additionally recorded 10 datasets from one subject (Subject 1) in the experiments, where the subject was not asked to press a button after error detection. We also conducted experiments in a scenario, in which a subject was asked to delay a motor response after error detection to avoid including features possibly used for ErrP detection. To this end we recorded three datasets from one subject (Subject 8) in both scenarios: (a) ErrP detections without motor response and (b) ErrP detections with delayed motor response. Here, three scenarios (motor response, no motor response, delayed motor response) were transferred to better interpret our assumption (see **Figure 3** and **Table 4**). However, these additional experiments only serve us as a preview for future work will need further experiments that were out of the focus of this work.

## 2 Methods

### 2.1 Subjects

Eight healthy subjects (four male and four female; 21.8 ± 2.4 ages; right-handed; students) participated in the experiments. All experiments were carried out in accordance with the approved guidelines. Experimental protocols were approved by the ethics committee of the department of Computer Science and Applied Cognitive Science of the Faculty of Engineering at the University of Duisburg-Essen. Written informed consent was obtained from all participants that volunteered to perform the experiments.

### 2.2 Experimental setup

**Figure 1:**
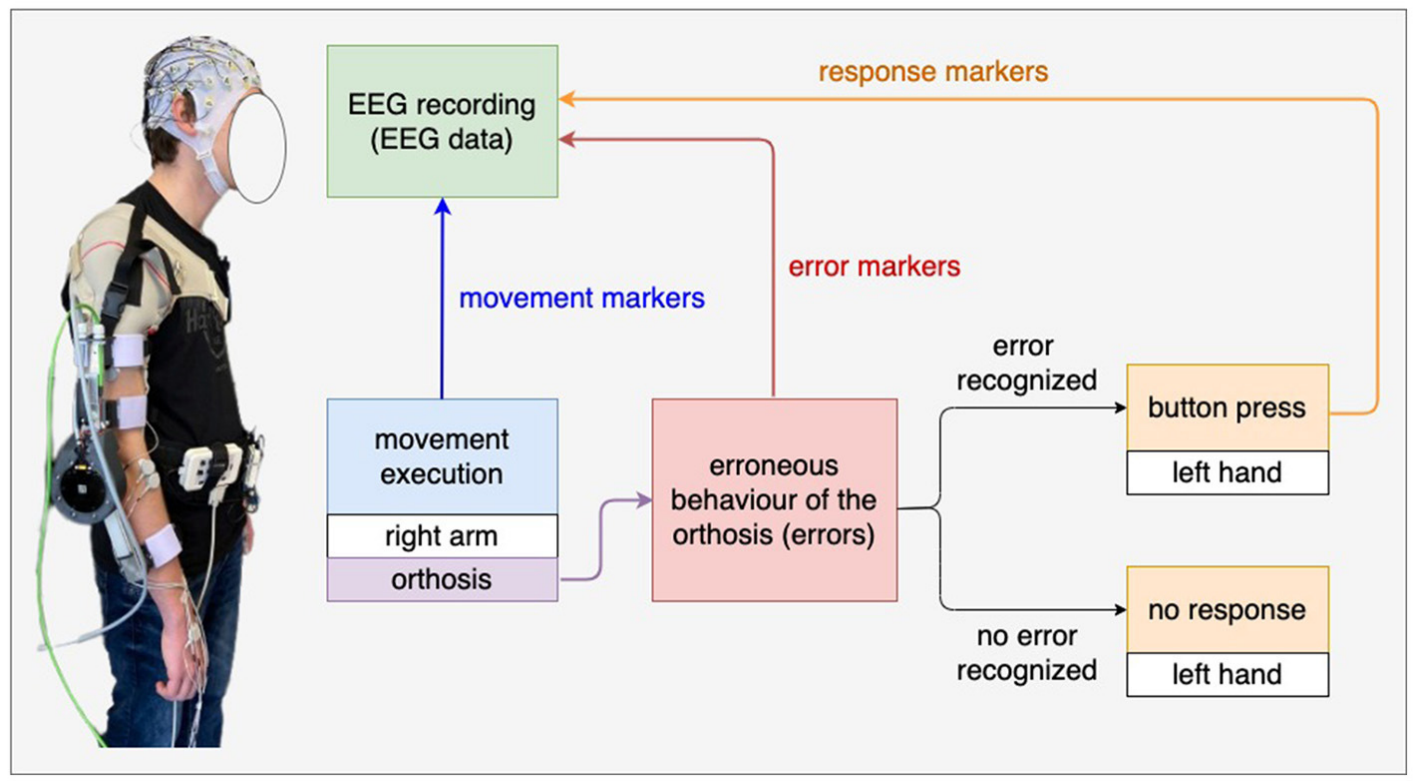
Experimental setup. The subjects wore an orthosis on their right arm and held a button with their left hand. In the main experiment, the subjects were instructed to press the button when they recognized tactile errors, i.e., incorrect behavior of the orthosis (e.g., the orthosis moved in the opposite direction of the intended movements of the subjects). We expect ErrPs when recognizing tactile errors.

**Figure 2:**
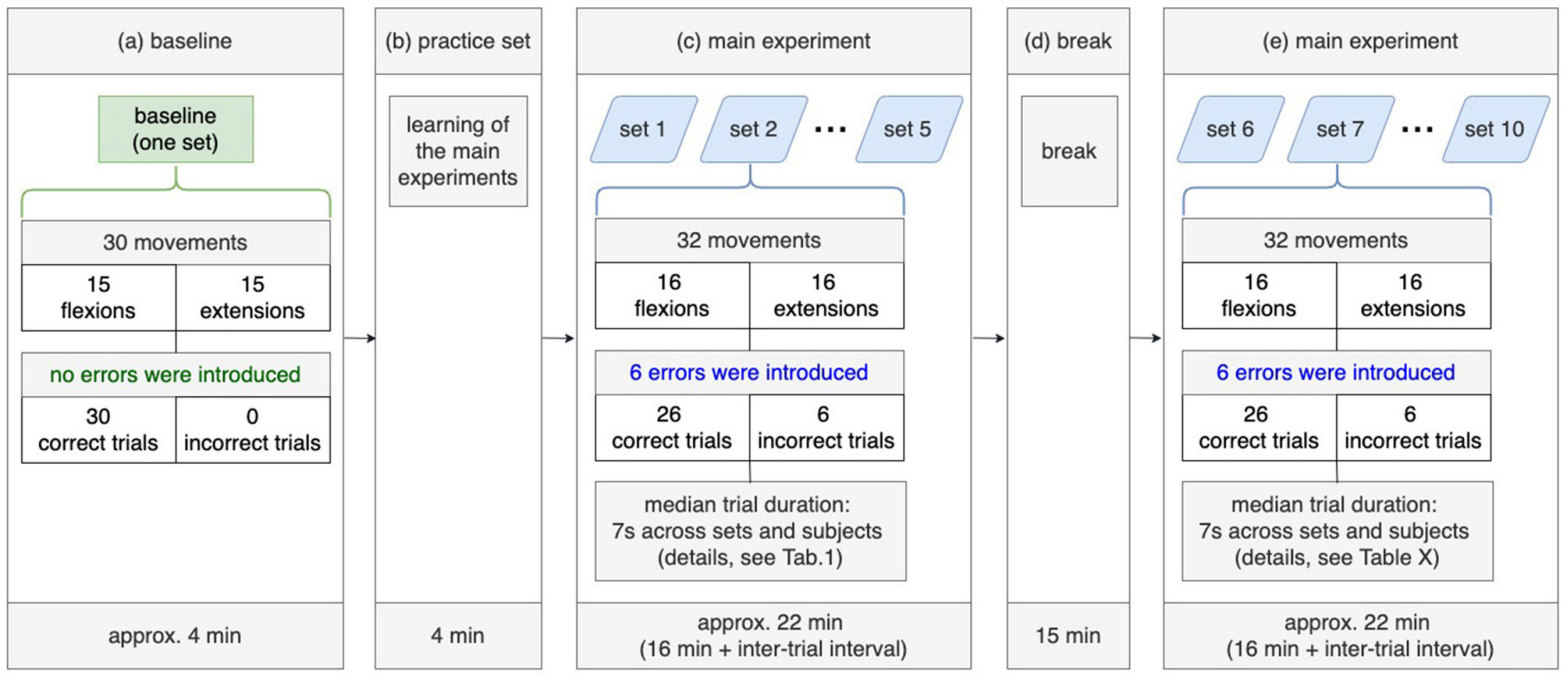
Experimental design. A baseline set without tactile errors **(a)** and the practice set including tactile errors **(b)** were recorded before the main experiment **(c, d)**, which divided into two parts: 5 sets **(c)** before the break and 5 sets after the break **(e)**.

Figure 1 shows the experimental setup. The subjects wore an orthosis on their right arm and held a button with their left hand. The orthosis was developed at the DFKI (details, see Kueper et al., 2023). The subjects performed arm movements consisting of flexions and extensions. We programmed the orthosis to move in the opposite direction of the subjects' intended movements with a certain probability for 0.25s. For example, subjects intended to extend their arm wearing the orthosis, but the orthosis briefly moved in the opposite direction of the intended movements. We hypothesized that such simulated malfunction of the orthosis, i.e., erroneous actions of the orthosis (errors) would elicit ErrPs. The subjects were asked to press a button when they felt that the orthosis was not functioning properly, i.e., when subjects felt erroneous actions of the orthosis. The button press was intended to serve as a response to the error detection. The responses (i.e., button presses) of subjects were sent to the EEG recording system so that the responses were written in the EEG data in real time (see Figure 1 response markers). The onset of movements (the onset of extension and flexion) and the onset of stimulated errors (i.e., incorrect orthosis behavior) were also sent to the EEG recording system (see Figure 1 movement markers and error markers, i.e., markers for error trials). Note that the orthosis position is denoted as −10° when the arm is fully extended, whereas the orthosis position is denoted as −90° when the arm is fully flexed. Malfunction of the orthosis (errors) are simulated to make the orthosis move in the opposite direction with the mean error position of −42° (flexion) and −58° (extension), respectively. The experiment procedure is described in detail in the next section (Section 2.3 and Figure 2).

### 2.3 Experimental design and procedure

Figure 2 shows the experimental design. Before the main experiment (see Figure 2a), subjects performed 30 movements (15 flexions, 15 extensions) without errors to familiarize themselves with the system (orthosis). After that, the practice set was performed to learn the experiment procedure of the main experiment (see Figure 2b). The main experiment consists of three parts: recordings of five sets, break, and recordings of further five sets (see Figures 2c–e).

In the main experiment, it was implemented that errors occur *randomly* with a probability of 20%. Two facts emerged from this: (a) errors can occur during the last movement (the last, i.e., 30th flexion and the 30th extension) and (b) 6 errors (i.e., simulated malfunction of the orthosis) occur within 30 arm movements (i.e., 24 correct trials and 6 incorrect trials). In the main study, we added two movement (one flexion, one extension) to avoid that the final trials (flexion and extension) did not contain simulated errors. Thus, the subject performed additional two movements after performing 30 movements. This results in two facts: (a) each set contained 32 movements (16 flexions and 16 extensions) and (b) the last movements cannot contain errors, since the errors were simulated within the first 30 arm movements. Finally, each set contained 26 correct trials and 6 incorrect trials (i.e., 6 errors).

We defined a trial as an extension movement or a flexion movement. We calculated the median value of trial duration since the trial duration varied depending on sets and subjects. The median trial duration was ~7 s across all subjects (details, see Table 1). The interval between trials (i.e., inter-trial interval) also varied across subjects, e.g., some subjects had a longer break between trials than others. The inter-trial interval ranged between 0.5 and 1 s per set. Thus, the task duration was between 4 and 5 min per set (32 trials for each set). We measured the task duration of 22 min for each recording phase (see Figures 2c, e) across all subjects. Hence, the duration of the main experiment was 44 min excluding the break between two recording phases, the practice set and the baseline experiment. According to the experimental design (Figure 2), the whole experiment should take 1 h and 7 min. However, the actual duration of the experiment was ~2 h, since we checked impedance and correct transfer of the markers in the EEG data file (see Figure 1) after each measurement (i.e., after recording of each set), which took ~5–10 min per set, and from 50 min to 1 h for all 10 sets per subject. Furthermore, the preparation of EEG and EMG measurements took between 1 and 2 h depending on subjects, where impedance was kept below 5 kΩ. Thus, the duration of the whole experiment including experiment preparation was between 3 and 4 h.

**Table 1:** Median trial duration for each subject and the mean value across subjects.

**Subject**	**Median trial duration (s)**
Subject 1	7.073
Subject 2	6.576
Subject 3	6.741
Subject 4	6.418
Subject 5	7.118
Subject 6	7.352
Subject 7	6.710
Subject 8	6.868
μ±σ	6.857 ± 0.309

μ±σ : mean ± standard deviation.

**Figure 3:**
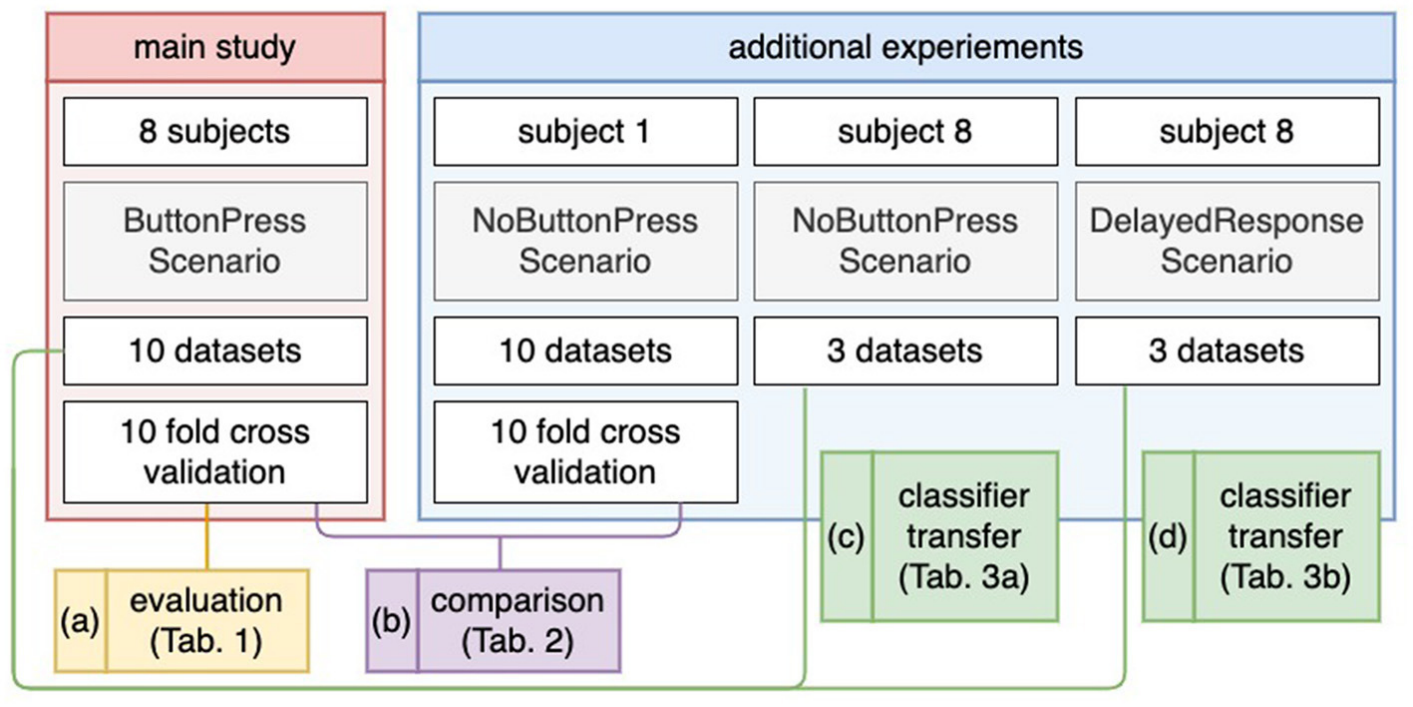
Evaluation design. For the main study **(a)**, a 10-fold cross validation was applied for evaluation. For the *NoButtonPress* scenario **(b)**, a 10-fold cross validation was also applied for evaluation, where the same subject (Subject 1) participated in the *ButtonPress* scenario and the *NoButtonPress* scenario on different days. For transfer learning **(c, d)**, the 10 datasets from the *ButtonPress* scenario (main study) was used to train a classifier. This trained classifier was used to evaluate the data from the *NoButtonPress* scenario **(c)** or *DelayedResponse Press* scenario **(d)**.

In addition to our main scenario described above, one subject (Subject 8) participated in two additional scenarios after the main scenario (*ButtonPress* scenario), in which (a) the subject was instructed not to give motor response after error detection (*NoButtonPress* scenario) and (b) the subject was asked to artificially delay a motor response (*DelayedButtonPress* scenario). Here, we recorded three datasets for the *DelayedButtonPress* scenario and three datasets for the *NoButtonPress* scenario, because the main study was expected to take 2 h. We started the following experiment sequences: *NoButtonPress* scenario, *DelayedButtonPress* scenario, and *ButtonPress* scenario. Indeed, the main study took 1 h 50 min. Recording more than 16 EEG data sets (3 h and 30 min without the preparation time) was not realistic. Note that 10 datasets from the main scenario (*ButtonPress* scenario) were used to train a classifier to evaluate the datasets from *DelayedButtonPress* scenario or *NoButtonPress* scenario (details, see Section 2.8.4 and Figure 3).

Another subject (Subject 1) participated in our main scenario (*ButtonPress* scenario) on one day and in the scenario where motor response was not required after error detection (*NoButtonPress* scenario) on another day. Here, we recorded 10 datasets for the *NoButtonPress* scenario just like for the main scenario (*ButtonPress* scenario). Due to the long duration of main study (at least 2 h and 30 min), it was not possible to record 10 datasets for each scenario (*ButtonPress* and *NoButtonPress*) at the same day from the same subject. The reason for this additional data acquisition was explained in Section 1.2.

### 2.4 Dataset

In the main experiment, we recorded 10 EEG datasets per subject. Each dataset contained 26 correct trials and 6 incorrect trials. Thus, we collected a total of 260 correct trials and 60 incorrect trials per subject. As mentioned earlier, we carried out experiments in two additional scenarios to test whether the ErrP-classification performance might be increased or decreased, e.g., without motor response. Thus, for one subject (Subject 8), we additionally recorded three datasets for error detection without motor response (*NoButtonPress*) and three datasets for error detection with delayed motor response (*DelayedButtonPress*). In total, for Subject 8, we recorded 10 datasets for error detection with motor response (*ButtonPress*), three datasets for error detection without motor response (*NoButtonPress*), and three datasets for error detection with delayed motor response (*DelayedButtonPress*). In the end, we also recorded 10 EEG datasets for one subject (Subject 1) on another day for error detection without motor response (*NoButtonPress*). In this way, we recorded 10 EEG datasets for error detection with motor response (*ButtonPress*) and 10 EEG datasets for error detection without motor response (*NoButtonPress*). For our main analysis, we used 10 EEG datasets with motor response from eight subjects.

### 2.5 Behavioral data acquisition

We recorded the behavioral data of subjects (e.g., response to errors) to analyze the accuracy of responses and response time per subject (see Figure 1).

### 2.6 EEG data acquisition

As mentioned earlier in Section, 2.4 we recorded EEG datasets from eight subjects. EEGs were continuously recorded using the actiCapSlim system (Brain Products GmbH, Munich, Germany), in which 64 active electrodes were arranged in accordance to an extended 10-20 system with reference at FCz. Impedance was kept below 5 kΩ. EEG signals were sampled at 500 Hz, amplified by two 32 channel BrainLiveAmp amplifiers (Brain Products GmbH, Munich, Germany), and filtered with a low cut-off of 0.1 Hz and high cut-off of 1 kHz. We sent EEG markers (labels) for relevant events to the EEG recording system to write the EEG markers into the continuous EEGs (see Figure 1): (a) movement onset for flexion, (b) movement onset for extension, (c) onset of simulated erroneous behavior of the orthosis (i.e., *ErrP* label), and (d) onset of button press to erroneous behavior of the orthosis (errors). In addition, we also sent a marker after the start of movement (flexion or extension) to obtain the *NoErrP* label, in case that the orthosis performed correctly.

### 2.7 Behavioral data analysis

We calculated response time, i.e., reaction time (RT) to error detection. We also calculated the number of undetected errors, i.e., false negatives (FN) and the number of incorrect responses, i.e., false positive (FP) responses. The analysis of the behavioral data was later used to exclude the epochs with erroneous response for both classes, e.g., button press for correct behavior of the orthosis or no button press for incorrect behavior of the orthosis (Details, see Section 2.8.1).

### 2.8 EEG data analysis

#### 2.8.1 EEG preprocessing

The EEG data was analyzed using a Python-based framework for preprocessing and classification (Krell et al., 2013) containing algorithms and external packages for feature extraction and classification [e.g., xDAWN (Rivet et al., 2009), pyRiemann (Barachant, 2015; Barachant et al., 2022)]. The continuous EEG signal was segmented into epochs from −0.1 to 1 s for each event type (correct/erroneous trial). Here, we excluded the epochs containing an incorrect response from EEG analysis. For example, epochs in which subjects pressed the button although the behavior of the orthosis was correct (false positive). On the other hand, we excluded epochs in which subjects did not press the button even though the behavior of the orthosis was incorrect (false negative). Hence, only the epochs with correct responses to errors (incorrect orthosis behavior) were labeled as *incorrect*, and the epochs with correct responses, i.e., no responses, to correct orthosis behavior were labeled as *correct*. All epochs were normalized to zero mean for each channel, decimated to 50 Hz, and band pass filtered (0.1–12 Hz).

#### 2.8.2 Feature extraction and classification

Features were extracted per subject. All datasets (i.e., 10 datasets) were concatenated per subject. The xDAWN spatial filter (Rivet et al., 2009) was used to enhance the signal-to-noise ratio. By applying the xDAWN the number of 64 physical channels was reduced to 7 pseudo channels. In this way, the signal-to-noise ratio for the positive class, i.e., *incorrect* class was maximized. All epochs were projected into the pseudo channels, i.e., 350 data points (7 channels × 50 data points) were obtained after applying xDAWN.

After applying xDAWN, we used a Riemmanian manifold approach (for review Yger et al., 2016; Congedo et al., 2017). First, we generated *extended epochs* (Barachant and Congedo, 2014) and obtained 14 pseudo channels (7·2 = 14 channels). We estimated a 14 × 14-dimensional covariance matrix across the 50 data points for each extended epoch. To this end, we used the shrinkage regularized estimator of Ledoit-Wolf (Ledoit and Wolf, 2004), which ensures that the estimated covariance matrices are positively defined. After estimating the covariance matrices, we approximated their Riemannian center of mass (Fréchet mean; Cartan, 1929) or often called geometric mean in BCI applications. This Riemannian center of mass was used as reference point to append a tangent space. All training and testing data were projected into this tangent space and vectorized using Mandel notation. Using Mandel notation, we reduced the symmetric 14 × 14-dimensional matrices into 105-dimensional feature vectors. After that, we normalized the feature vectors. For classification, we used a linear Support Vector Machine (SVM; Cortes and Vapnik, 1995; Mangasarian and Musicant, 1999). We optimized the cost parameter of the SVM (i.e., regularization constant; Schölkopf et al., 2000). To this end, the hyperparameter *C* of the SVM was selected from {10^−6^, 10^−5^, 10^−4^, 10^−3^, 10^−2^, 10^−1^, 1} using a five-fold stratified cross validation, in which the correct (*NoErrP*) and incorrect (*ErrP*) classes were weighted as 1:2.

#### 2.8.3 Event-related potential analysis

For ERP analysis, we analyzed the EEG data using EEGLAB^1^. For preprocessing, the raw EEGs were downsampled to 250 Hz, re-referenced to an average reference, and filtered between 0.1 and 15 Hz. The *FCz* channel, used as a reference in the EEG recording, was recalculated as an EEG channel for ERP analysis. After preprocessing, independent component analysis (ICA) was applied for artifact removal. We used Infomax ICA to remove artifacts (e.g., muscle, eye blinks, or eye movements) by subtracting ICA components containing eye artifacts. After artifact removal, EEG data were segmented into epochs from 0.1 to 1 s after each event type (correct/incorrect). Epochs were averaged within each event type with a baseline correction (-0.1 s until stimulus onset) per subject. For calculation of grand average ERP, the ERPs of all subjects were averaged.

#### 2.8.4 Evaluation

Figure 3 shows the evaluation design for our study. For evaluation, we used two labels: correct behavior of the orthosis (*NoErrP*) and incorrect behavior of the orthosis (*ErrP*). As mentioned earlier, we concatenated the epochs of the 10 datasets and obtained a total of 260 correct trials and 60 incorrect trials per subject. A ten fold stratified cross validation was applied on the concatenated datasets per subject (see Figure 3a). Thus, we obtained the classification performance for each subject. For performance metric, we used a balanced accuracy, i.e., the arithmetic mean of true positive rate (TRP) and true negative rate (TNR). We also additionally reported TPR (recall), TNR, F measure, and precision (Details, see Section 3.2). Note that the positive class stands for incorrect behavior of the orthosis and negative class stands for correct behavior of the orthosis. For transfer learning, we trained a classifier based on the 10 datasets recorded in the scenario (*ButtonPress* scenario), in which motor response was required after error detection. This trained classifier was used to test the three datasets and three datasets, respectively recorded in the scenario (*NoButtonPress* scenario) where motor response was not required after error detection (see Figure 3c) or in the scenario (*DelayedButtonPress* scenario) where delayed response was required after error detection (see Figure 3d). For transfer learning, as mentioned earlier, we evaluated datasets from both scenarios for one subject (Subject 8). For the evaluation of *NoButtonPress* scenario, the same evaluation process was used for the evaluation of *ButtonPress* scenario (main study) to compare the two scenarios *ButtonPress* and *NoButtonPress* (see Figure 3b), where the same subject (Subject 1) participated in both scenarios on different days.

## 3 Results

### 3.1 Behavior data

**Figure 4:**
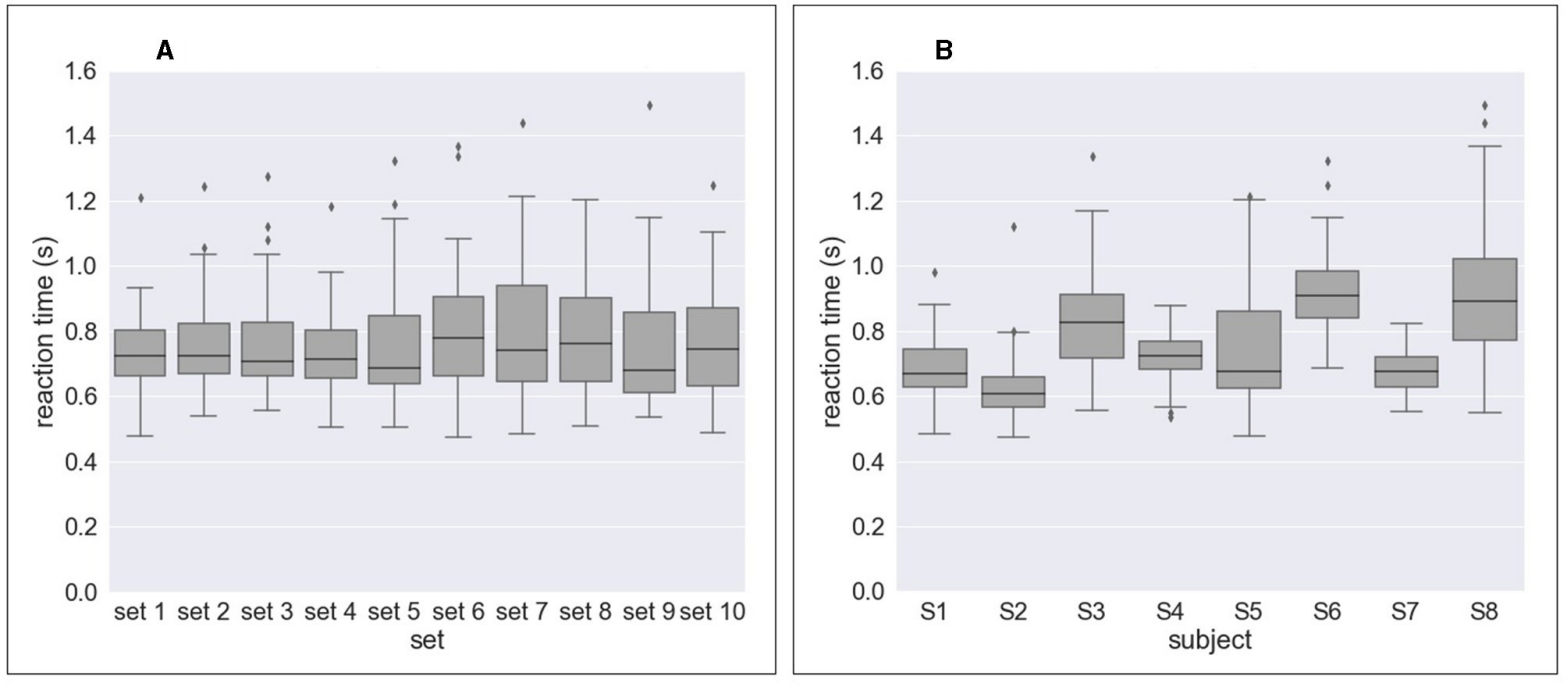
Response time to erroneous behavior of the orthosis (i.e., reaction time) for all sets and all subjects. **(A)** Reaction time per set across eight subjects. **(B)** Reaction time per subject across 10 sets.

**Figure 5:**
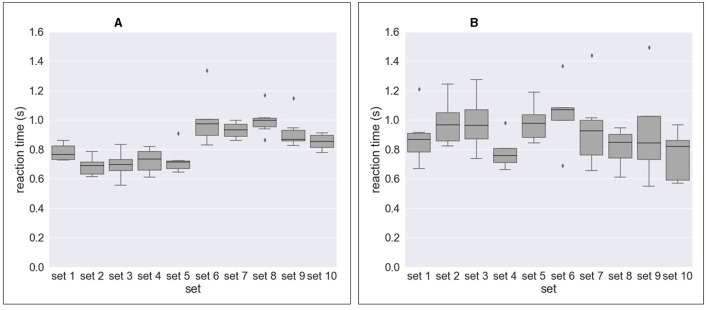
Response time to erroneous behavior of the orthosis (i.e., reaction time) for Subject 3 and Subject 8 as example. **(A)** Reaction time (subject 3). **(B)** Reaction time (subject 8).

**Figure 6:**
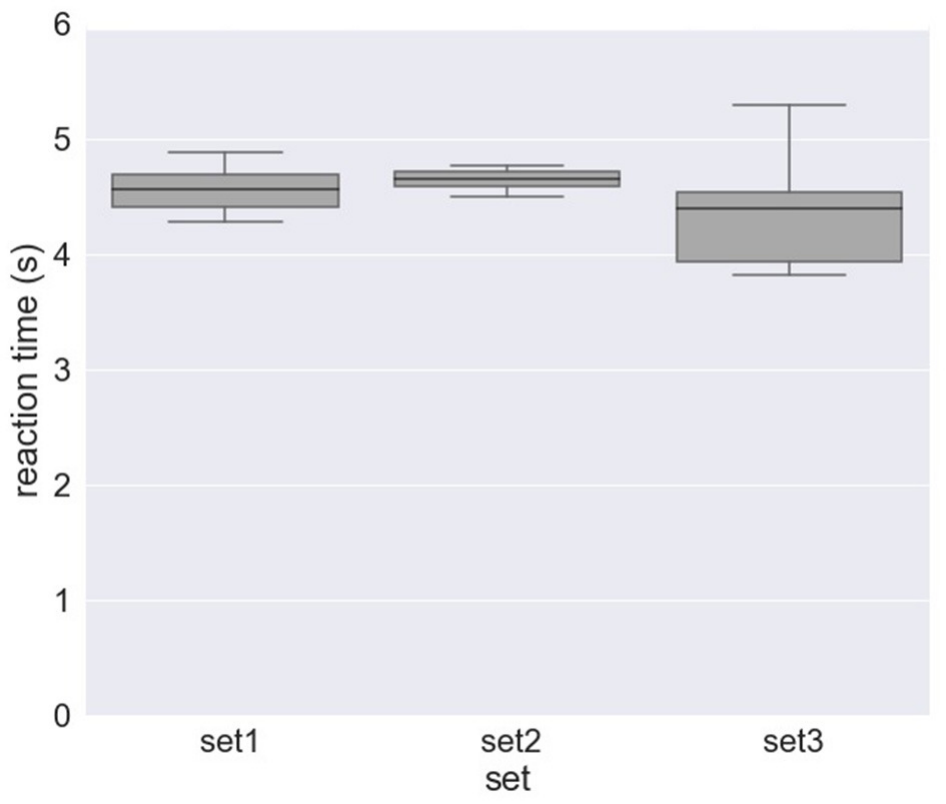
Response time to erroneous behavior of the orthosis (i.e., reaction time) for Subject 8 in the experiment, where the subject was asked to delay his motor response after error detection (*DelayedButtonPress* experiment).

Figure 4 and Table 2 shows the results of response time, i.e., reaction time (RT) to errors. Figure 4A and Table 2A show the median value of reaction time for each set across eight subjects whereas Figure 4B and Table 2A show the median value of reaction time for each subject across 10 sets. We obtained a mean of 0.746 s RT across all subjects (Table 2A) and a mean of 0.728 s RT across all sets (Table 2B). However, as shown in Figure 4A, a high variation in reaction time between subjects was observed for all sets. Similarly, the variation in reaction time between sets was observed for all subjects except for Subject 4 and Subject 7 (Figure 4B). For example, the reaction time after half of the experiments (from Set 6) was longer in Subject 3 (Figure 5A), whereas the reaction time was highly different between sets for Subject 8 (Figure 5B). Figure 6 shows the results of response time, i.e., reaction time (RT) to errors in the experiment, in which the subject (subject 8) was asked to delay his motor response after error recognition. We obtained a mean of 4.54 s RT across all sets for this subject.

**Table 2:** Results of response time (RT).

**(A) Median RT for each subject across datasets**		**(B) Median RT for each set across subjects**	
**Subject**	**Median reaction time (s)**	**Dataset**	**Median reaction time (s)**
Subject 1	0.668	Set 1	0.724
Subject 2	0.608	Set 2	0.728
Subject 3	0.826	Set 3	0.701
Subject 4	0.720	Set 4	0.714
Subject 5	0.674	Set 5	0.696
Subject 6	0.910	Set 6	0.796
Subject 7	0.676	Set 7	0.738
Subject 8	0.890	Set 8	0.758
		Set 9	0.684
		Set 10	0.742
μ±σ	0.746 ± 0.113	μ±σ	0.728 ± 0.032

(A) Median reaction time for each subject across 10 datasets. (B) Median reaction time for each set across 8 subjects. μ±σ : mean ± standard deviation.

We also analyzed the accuracy of the response to errors. We have calculated the number of undetected errors, i.e., false negatives (FN). A total of 471 errors were detected from 480 errors (6 errors × 10 sets × 8 subjects). This results in an average error detection accuracy of 98.82% for all subjects. In other words, we obtained a mean of 1.88% FN across all subjects. We also calculated the number of incorrect responses, i.e., false positive (FP) responses. (i.e., the button was pressed even though there was no error). We obtained a mean of 0.83% FP for all subjects.

### 3.2 EEG data

#### 3.2.1 EEG classification performance

Table 3 shows ErrP-classification performance. We obtained high ErrP classification performance for all subjects. The mean value of TNR was slightly higher compared to TPR, i.e., the false positive rate (1-TNR) was less than the false negative rate (1-TPR). We also found more variability between subjects for TPR than TNR.

**Table 3:** ErrP-classification performance.

**Subject**	**bACC**	**TPR**	**TNR**	**F measure**	**Precision**
Subject 1	0.987	0.986	0.989	0.971	0.960
Subject 2	0.979	0.967	0.992	0.966	0.971
Subject 3	0.962	0.933	0.992	0.946	0.971
Subject 4	0.998	1.000	0.997	0.993	0.975
Subject 5	0.973	0.955	0.992	0.961	0.973
Subject 6	0.973	0.955	0.992	0.961	0.973
Subject 7	0.991	0.986	0.996	0.984	0.985
Subject 8	0.934	0.889	0.982	0.899	0.918
Mean ± SEM	0.975 ± 0.007	0.959 ± 0.013	0.992 ± 0.002	0.960 ± 0.010	0.966 ± 0.007

Mean and standard error of mean (SEM) are reported. Note that the positive class stands for erroneous behavior of the orthosis (*ErrP* label). TPR, true positive rate (called recall); TNR, true negative rate; bACC, balanced accuracy [(TPR+TNR)/2]).

Table 4 shows ErrP-classification performance for transfer learning. As mentioned earlier, we performed experiments in two additional scenarios to evaluate whether ErrP-classification performance might be increased or decreased, e.g., without motor response. We trained a classifier using the datasets recorded in the main scenario, in which motor response was required after error recognition (*ButtonPress*). This trained classifier was used to test (a) the datasets recorded in the scenario, in which the subjects were instructed not to give motor response after error recognition (*NoButtonPress*) or (b) the datasets recorded in the scenario, where subjects were asked to artificially delay motor response after error detection (*DelayedButtonPress*).

**Table 4:** ErrP-classification performance for two scenarios.

	**bACC**	**TPR**	**TNR**	**F measure**	**Precision**
**(A) Motor response after error detection (** ***ButtonPresss*** **)**					
Subject 1	0.987	0.986	0.989	0.971	0.960
**(B) No motor response after error detection (** ***NoButtonPresss*** **)**					
Subject 1	0.991	0.986	0.997	0.985	0.987

(A) motor response after error detection (*ButtonPresss*) and (B) no motor response after error detection (*NoButtonPresss*; Subject 1).

Mean and standard error of mean (SEM) are reported. Note that the positive class stands for erroneous behavior of the orthosis (*ErrP* label). TPR, true positive rate (called recall); TNR, true negative rate; bACC, balanced accuracy [(TPR+TNR)/2]).

As shown in Table 4A, we still observed a high TPR, but a slightly lower TNR when transferring from the scenario with motor response to the scenario without motor response. That means, the rate of error detection (ErrP detection) was still high, but there were many false alarms, i.e., the false positive rate was high (1-TNR). In contrast, we observed a very low TPR, but a high TNR when transferring from the scenario with motor response to the scenario with delayed motor response (see Table 5B). That means, the false alarm (false positive rate) was not high, but the rate of error detection (ErrP detection) was very low.

**Table 5:** ErrP-classification performance for scenario transfer (Subject 8).

	**bACC**	**TPR**	**TNR**	**F measure**	**Precision**
**(A) Training: motor response after error detection, Test: no motor response after error detection**					
Subject 8	0.913	1.000	0.827	0.727	0.571
**(B) Training: motor response after error detection, Test: delayed response after error detection**					
Subject 8	0.747	0.533	0.962	0.615	0.727

Mean and standard error of mean (SEM) are reported. Note that the positive class stands for erroneous behavior of the orthosis (*ErrP* label). TPR, true positive rate (called recall); TNR, true negative rate; bACC, balanced accuracy [(TPR+TNR)/2]).

Table 5 shows the comparison between two scenarios (motor response after error detection vs. no motor response after error detection) in ErrP-classification performance in the same subjects (subject 1). Note that the datasets were not recorded on the same day. We achieved high ErrP-classification performance for both scenarios. That means, we obtained high ErrP-classification performance in the scenario, in which motor response was not required after error detection.

#### 3.2.2 Event-related potential analysis

**Figure 7:**
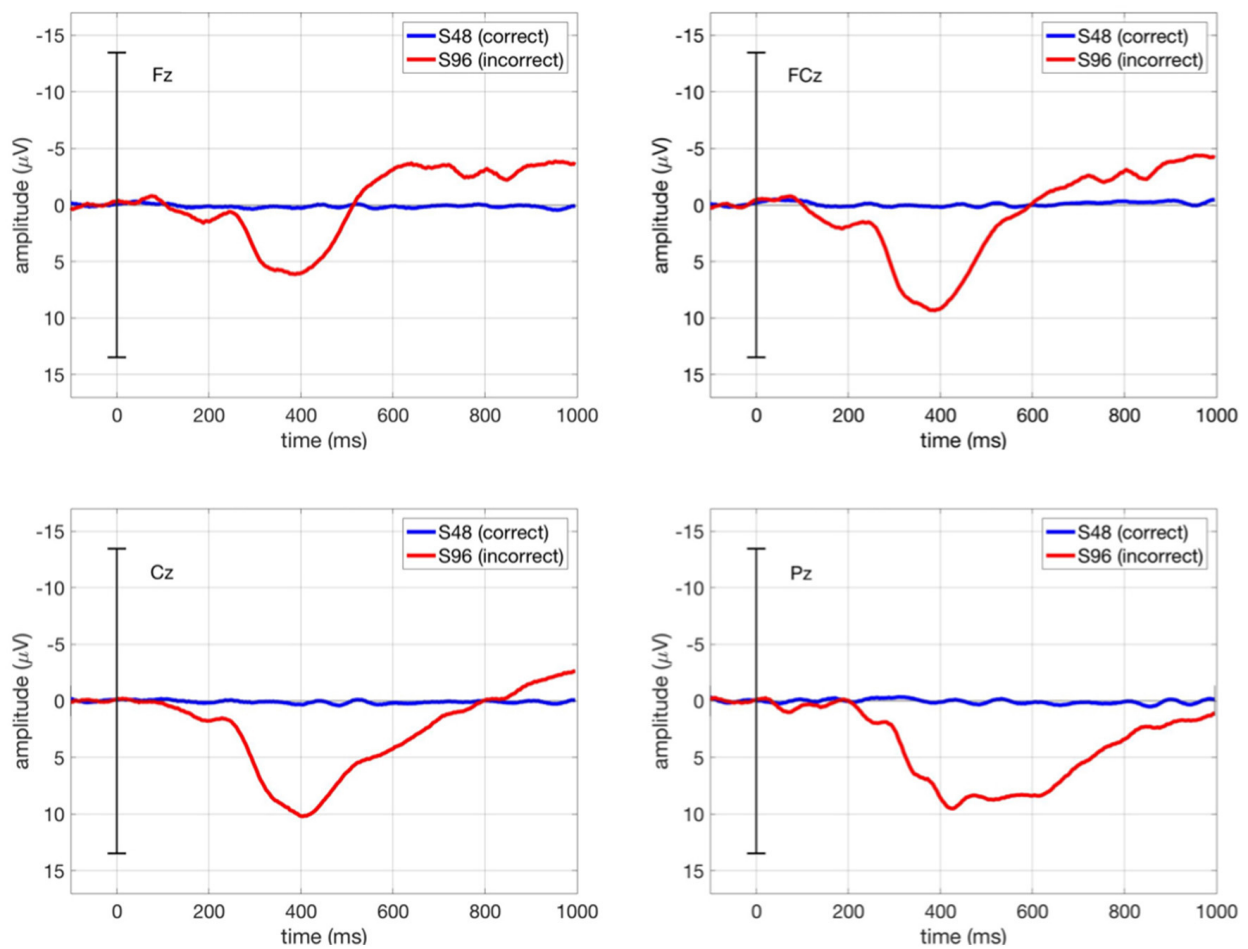
Grand average ERPs in our main study (*ButtonPress* scenario) at Fz, FCz, Cz, and Pz. The blue curve stands for the ERPs averaged over all correct trials (S48), whereas the red curve stands for the ERPs averaged over all incorrect trials (S96). Grand average ERP stands for the ERPs averaged across all subjects for each condition (correct/incorrect).

Figure 7 shows the grand average ERPs in our main study (*ButtonPress* scenario) at Fz, FCz, Cz, and Pz, in which the ERPs averaged over all trials per condition (correct/incorrect) for each subject were in turn averaged over all subjects. We observed the first negativity around 250 ms followed by a positivity between 300 and 500 ms and a further negativity around 600 ms. Here, the ERP shape (i.e., temporal sequences of ErrPs) is identical to other ErrP studies (e.g. Chavarriaga et al., 2012). However, the first negativity was suppressed due to the strong positivity in its minus polarity. As expected from ERP pattern evoked by interaction errors, the first and second negativity was smaller at Cz and Pz than at Fz and FCz.

**Figure 8:**
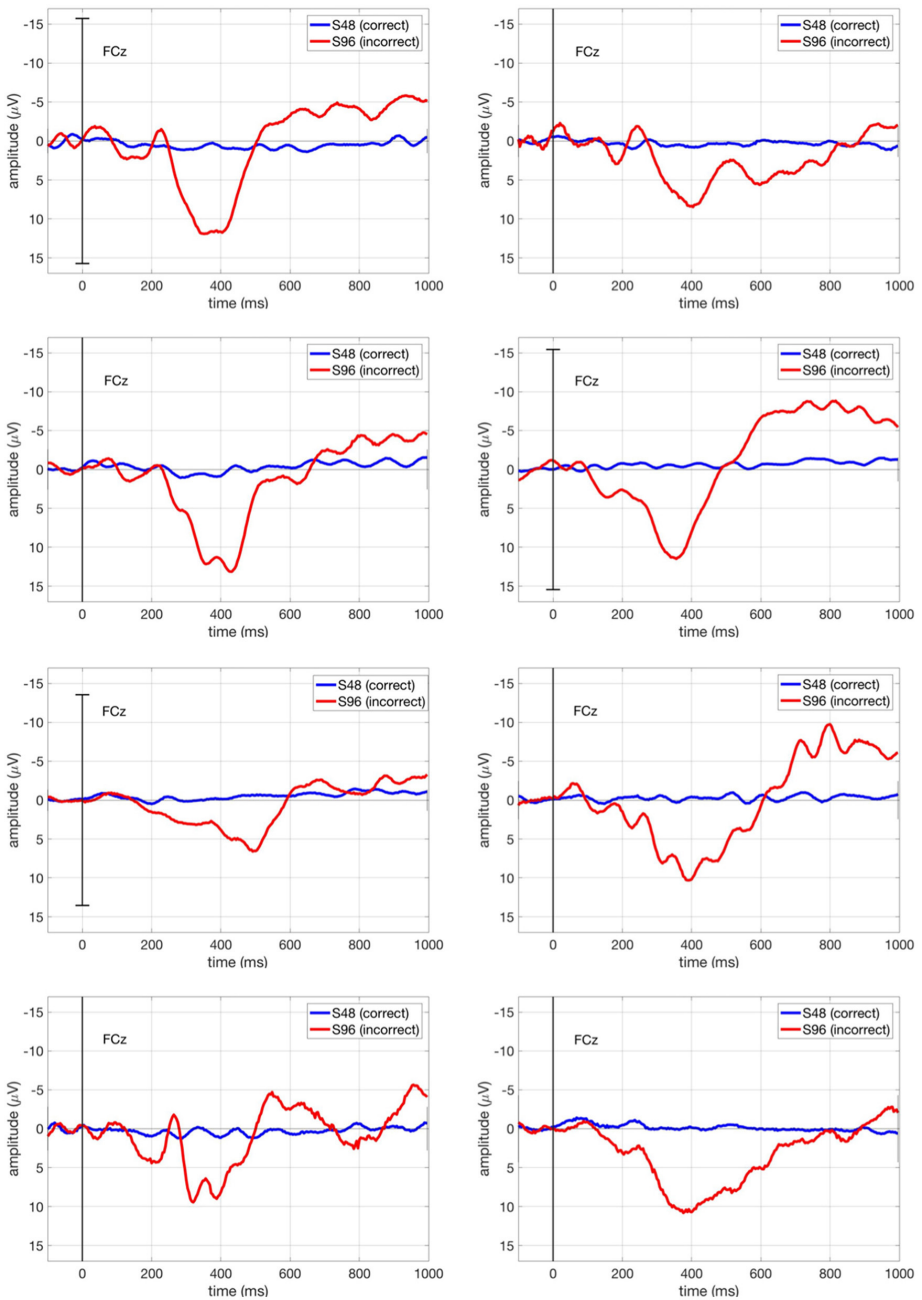
ERPs in our main study (*ButtonPress* scenario) for each subject at FCz. The blue curve stands for the ERPs averaged over all correct trials (S48), whereas the red curve stands for the ERPs averaged over all incorrect trials (S96).

Figure 8 shows the ERP curve averaged across all correct trials (S48, blue curve) and all incorrect trials (S96, red curve) for each subject at FCz, in which we expect maximum ErrPs at fronto-central region (FCz). We observed that Subject 7 showed most clearly the expected classic ErrP form (first negativity followed by positivity and second negativity).

**Figure 9:**
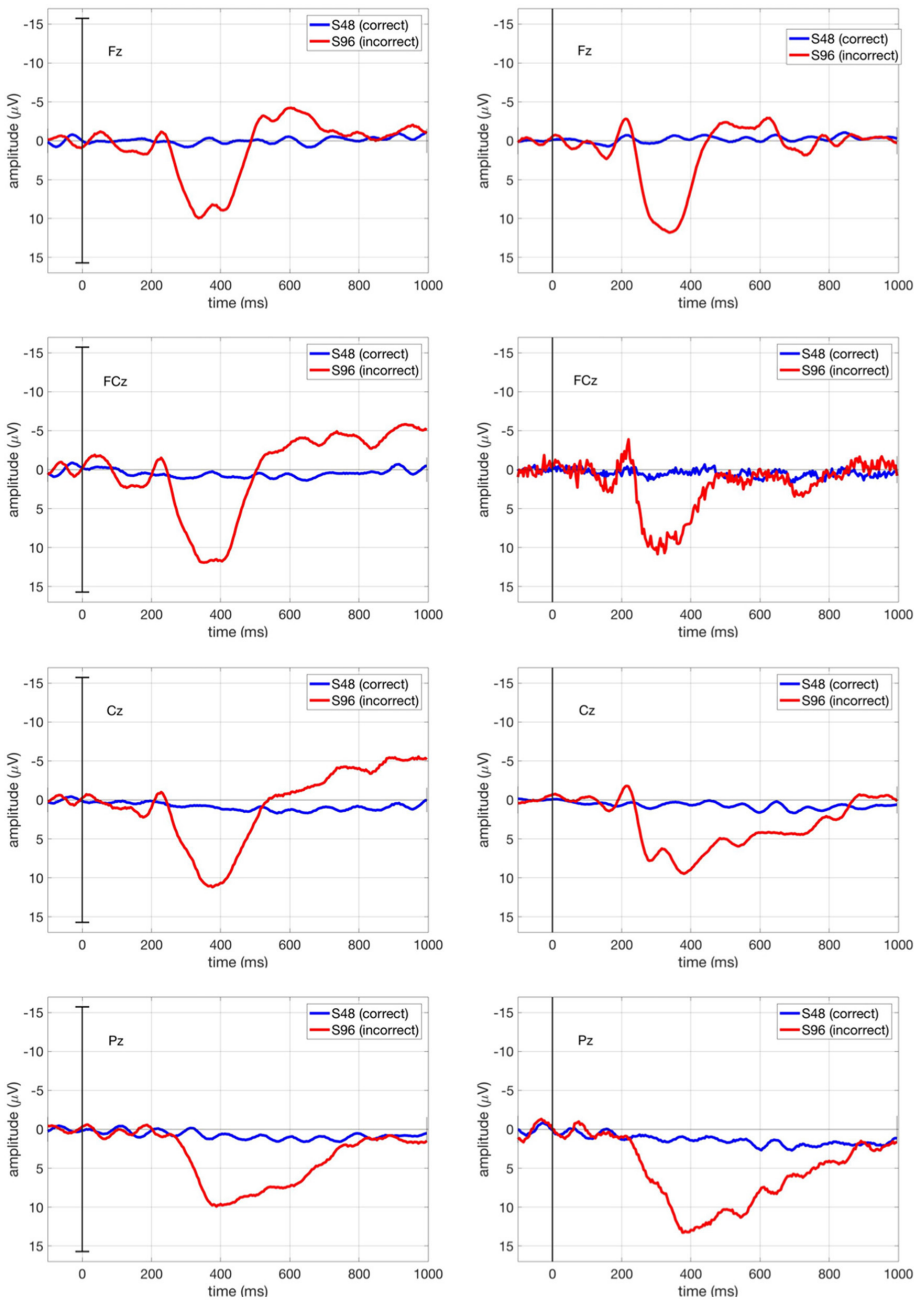
ERPs for Subject 1 at Fz, FCz, Cz, and Pz in both *ButtonPress* scenario and *NoButtonPress* scenario. The blue curve stands for the ERPs averaged over all correct trials (S48), whereas the red curve stands for the ERPs averaged over all incorrect trials (S96).

Figure 9 shows the ERP curve averaged across all correct trials (S48, blue curve) and all incorrect trials (S96, red curve) for Subject 1 in comparison for both *ButtonPress* scenario and *NoButtonPress* scenario. The ErrP shape is similar in both scenarios.

## 4 Discussion

In our study, we developed a scenario in which tactilely triggered ErrPs were examined for tactilely perceived misbehavior of an orthosis. The behavioral results showed that interaction errors mediated by erroneous behavior of the orthosis via the tactile channel were detected with a high accuracy (98.82%) by all subjects. Our EEG results showed that it is feasible to detect ErrPs, which are elicited when interaction errors are detected *only* via the tactile channel without visual, auditory, or tactile cues. Thus, our results revealed that it was also feasible to detect tactile-based ErrPs without combining them with visual error recognition.

Further, the comparison in ErrP-classification performance between two scenarios (motor response after error detection and no motor response after error detection) revealed that ErrP detection is also feasible without making use of features from motor activity (Table 5). Note that we performed this evaluation on one subject (10 datasets recorded from the scenario *motor response after error detection* vs. 10 datasets recorded from the scenario *no motor response after error detection*) and the results are therefore preliminary. These results are consistent with our previous studies (Kim et al., 2017, 2020a,b), in which subjects observed the robot's behavior while interacting with the robot and did not perform a motor response (e.g., press a button) after detecting erroneous actions of a robot.

Furthermore, reaction-time analysis show that the mean response time across all subjects was 746 ms. This means that the motor response occurred very late, which can only affect the last part of the ERP evoked by errors (i.e., the second negativity). Moreover, response time is not very strongly time locked to the error, as reaction-time analysis has shown. For this reason, we expect that the effect of motor response should be small. As mentioned in Section 1, in many ErrP studies, the recognition of errors elicited ErrPs without any motor response. In most studies, errors were recognized visually while observing/monitoring errors (visual feedback) without a motor response, and this visual recognition of errors (error monitoring) elicited ErrPs. Furthermore, there was an ErrP study (Chavarriaga et al., 2012) conducted with motor response (*KeyPress* after error recognition) and without motor response (error monitoring) using visuo-tactile stimuli. In this study, ErrPs were evoked under both conditions, and the ERP shape was similar under both conditions. Although our study cannot prove that motor activity does not contribute to the observed ERP curve, the additional experiment with motor response in subject 1 showed that a similar ERP shape was observed in case of no motor response. We want to emphasize that we required motor response to ensure that the subjects recognized tactile-based errors.

An interesting result is that ErrP-classification performance was only slightly reduced (91%) when we applied the classifier trained on the datasets containing interaction errors with motor response to test the datasets containing interaction errors without motor response (Table 4A). These results suggest that the features used for motor potential detection are not very relevant for ErrP detection. In contrast, the ErrP-classification performance was substantially reduced (74%) when we used the classifier trained on the datasets containing interaction errors with motor response to test the datasets containing interaction errors with delayed motor response (Table 4B). These findings suggest that features used from motor related activity could have a negative effect on ErrP detection when the motor response was artificially delayed by subjects. Again, these results are preliminary and need further studies. We performed theses tests to develop more ideas into future possible experiments.

Another interesting preliminary finding is that we observed a very high true positive rate (100%) and an increased false positive rate (17%) when we transferred the classifier trained on the datasets containing interaction errors with motor response to the datasets containing interaction errors without motor response (Table 4A). Here, we assumed that the effect of maximizing the positive class (incorrect class, i.e., ErrP label) during feature extraction (details, spatial filtering in Section 2.8.2) might become stronger when the test data did not include features detecting motor potentials. For this reason, the true positive rates could be increased whereas the false positive rate could be decreased. On the other hand, we observed a very low true positive rate (53%) and a low false positive rate (4%) when we transferred the classifier trained on the datasets containing interaction errors with motor response into the datasets containing interaction errors with delayed motor response (Table 4B). Again, the results suggest that features used for motor potential detection could have a negative impact on ErrP detection when the motor response was artificially delayed by subjects.

However, we studied only one subject for transfer learning. Therefore, the results for transfer learning are preliminary and it is necessary to investigate this systematically with more subjects in future work. We have shown with our preliminary results that our main scenario that allows us to prove that subjects detect tactilely induced errors, can be adapted to gain more insight into the correlation between different EEG activities by making modifications such as those for the *NoButtonPress* or *DelayedButtonPress* conditions. Thus, our preliminary findings from classifier transfer and adaptation of response situation opens up interesting research questions for future experiments worth it to be investigated.

The ERP results (Figure 7) indicates that ErrPs could be also shown at frontal region (e.g., Fz) when errors were introduced by tactile stimuli. These results are consistent with the findings of Chavarriaga et al. (2012), which showed a similar ERP morphology (first negativity followed positivity and a further negativity) at frontal region (Fz) when errors were introduced by visuo-tactile stimuli. In our study, the positivity was stronger than in other ErrP studies (review see Chavarriaga et al., 2014). We first assumed that the task-relevant event (i.e., the button press when recognizing errors instead of passively observing errors) might induce a stronger positivity (i.e., P300) in our study. However, the test with one subject (Subject 1) in the *NoButtonPress* scenario compared to the data recorded for this subject in the *ButtonPress* scenario did not support this hypothesis. We found no strong differences for this one subject under the response and the no response condition (see Figure 9). This interesting preliminary result opens up future research questions on the processing of tactile stimuli. Further studies are needed in the future.

In recent years, there has been increased interest and impact of the human-in-the-loop approach to human-robot interaction and also to tele-rehabilitation (e.g., therapist-in-the-loop in assisted as needed approach). Here, tactile feedback (tactile stimulation or force feedback) has a great importance for a better cooperation with the users and a better support of the patients. Therefore, in the future, we want to investigate errors or resistance that are received via tactile channels from users or patients, which can occur during human-robot interaction in our robotic supported (tele-) rehabilitation scenarios.

## Data availability statement

The datasets presented in this study can be found in online repositories. The names of the repository/repositories and accession number(s) can be found at: https://zenodo.org/record/8345429.

## Ethics statement

The studies involving humans were approved by Ethics Committee of the Department of Computer Science and Applied Cognitive Science of the Faculty of Engineering at the University of Duisburg-Essen. The studies were conducted in accordance with the local legislation and institutional requirements. The participants provided their written informed consent to participate in this study.

## Author contributions

SK: Conceptualization, Investigation, Methodology, Validation, Visualization, Writing—original draft. EK: Conceptualization, Funding acquisition, Project administration, Resources, Supervision, Writing—review & editing.

## Footnotes

^1^ https://sccn.ucsd.edu/eeglab/index.php
